# Role of DDX3 in the pathogenesis of inflammatory bowel disease

**DOI:** 10.18632/oncotarget.23323

**Published:** 2017-12-15

**Authors:** Saritha Tantravedi, Farhad Vesuna, Paul T. Winnard, Marise R. Heerma Van Voss, Paul J. Van Diest, Venu Raman

**Affiliations:** ^1^ Department of Radiology and Radiological Science, Johns Hopkins University, School of Medicine, Baltimore, MD, USA; ^2^ Department of Pathology, University Medical Center Utrecht, Utrecht, The Netherlands; ^3^ Department of Oncology, Johns Hopkins University, School of Medicine, Baltimore, MD, USA

**Keywords:** DDX3, inflammatory bowel disease, colorectal cancer, MMP, small molecule inhibitor

## Abstract

When crypt stem cells of the gastrointestinal tract become injured, the result is increased synthesis of pro-inflammatory cytokines and matrix metalloproteinases by their progeny – the colonic epithelium. Chronic inflammation of the gastrointestinal tract is a characteristic of inflammatory bowel disease, which includes Crohn’s Disease and Ulcerative Colitis. In our ongoing investigation to decipher the characteristic functions of a RNA helicase gene, DDX3, we identified high DDX3 expression by immunohistochemistry of colon biopsy samples, which included chronic/mild Morbus Crohn, active Morbus Crohn, Chronic/mild Colitis Ulcerosa and active Colitis Ulcerosa in epithelium and stromal compartments. We used a small molecule inhibitor of DDX3, RK-33, on two human colonic epithelial cell lines, HCEC1CT and HCEC2CT and found that RK-33 was able to decrease expression of MMP-1, MMP-2, MMP-3, and MMP-10. Moreover, forced differentiation of a human colonic cancer cell line, HT29, resulted in decreased DDX3 levels, indicating that DDX3 contributes to the modulation of colonic epithelium differentiation. In conclusion, our results revealed novel functions of DDX3 in inflammatory bowel disease and indicate a potential for using RK-33 as a systemic therapy to promote not only differentiation of transformed colonic epithelium but also to reduce MMP expression and thus elicit a decreased inflammatory response.

## INTRODUCTION

The colonic epithelium provides a necessary barrier to protect the internal milieu of the intestine from the potentially injurious luminal environment [1, 2]. However, crypt stem cells are susceptible to injury that can lead to increased synthesis of inflammatory cytokines and matrix metalloproteinases by their progeny, the colonic epithelium [3–6]. Persistence of these conditions contributes to the development of inflammatory associated bowel diseases, such as ulcerative colitis (UC) and Crohn’s disease (CD). UC and CD are important public health problems, with increasing incidence rates, high morbidity and high patient costs [7, 8]. These inflammatory bowel diseases (IBD) are due in part to an autoimmune response against the epithelium that lines the intestinal surface [9, 10]. Whereas UC presents exclusively in the large intestine, CD can manifest itself in the entire digestive tract [11]. Recent advances have been made in understanding the pathogenesis of IBD. However, gaps in our knowledge persist and the exact etiology of both UC and CD remains elusive [10]. For example, it is not well understood why, in a substantial group of patients, the disease persists over time. Along these lines, general changes in the gut epithelium are known to have a major effect in the development and progression of IBD and yet, current treatment strategies mainly focus on targeting the overactive immune system [12, 13]. However, even after long-term treatment with severe immunosuppressive agents, many of these patients eventually require colectomies and IBD remains associated with an increase in intermediate and long-term mortality of 10% and 50% for UC and CD, respectively [14]. This has spurred further research and insights into the complex interactions between the immune system, abdominal microflora, and the intestinal epithelium all of which contribute to the pathogenesis of IBD. Such studies have provided evidence that enterocytes (columnar epithelial cells) trigger and enhance a local immune response by the production of proinflammatory cytokines and matrix metalloproteinases (MMPs) [15, 16].

MMPs are a family of 24 zinc- dependent endopeptidases, which are transcriptionally upregulated in response to proinflammatory cytokines [17]. Increased amounts of MMP-1, MMP-2, MMP-3, MMP-7, MMP-9, MMP-10, MMP-12, and MMP-13 are produced by human colonic epithelial cells in IBD patients [15]. As a corollary, it is important to note that chronic inflammation is a major contributor to gastrointestinal carcinogenesis and patients suffering from IBD are at higher risk to develop colonic neoplasia [18], and colorectal cancer accounts for 15% of all UC-related deaths [19]. Consequently, MMPs, and the associated inflammation, are one of the targets for the treatment of IBD and associated colorectal cancer [20–24].

In the present study, we have shown that DDX3 expression levels are elevated in IBD cases with active inflammation and not only in epithelial cells but also in the stromal compartment. We have rationally designed and synthesized a small molecule inhibitor of DDX3, RK-33, and our data demonstrate that this drug has a high potential of being used as a systemic therapy for IBD as treating human colonic epithelial cells with RK-33 reduces the expression of MMPs, which are important in the development of chronic inflammation.

## RESULTS

### DDX3 expression levels are increased in IBD

**Figure 1:**
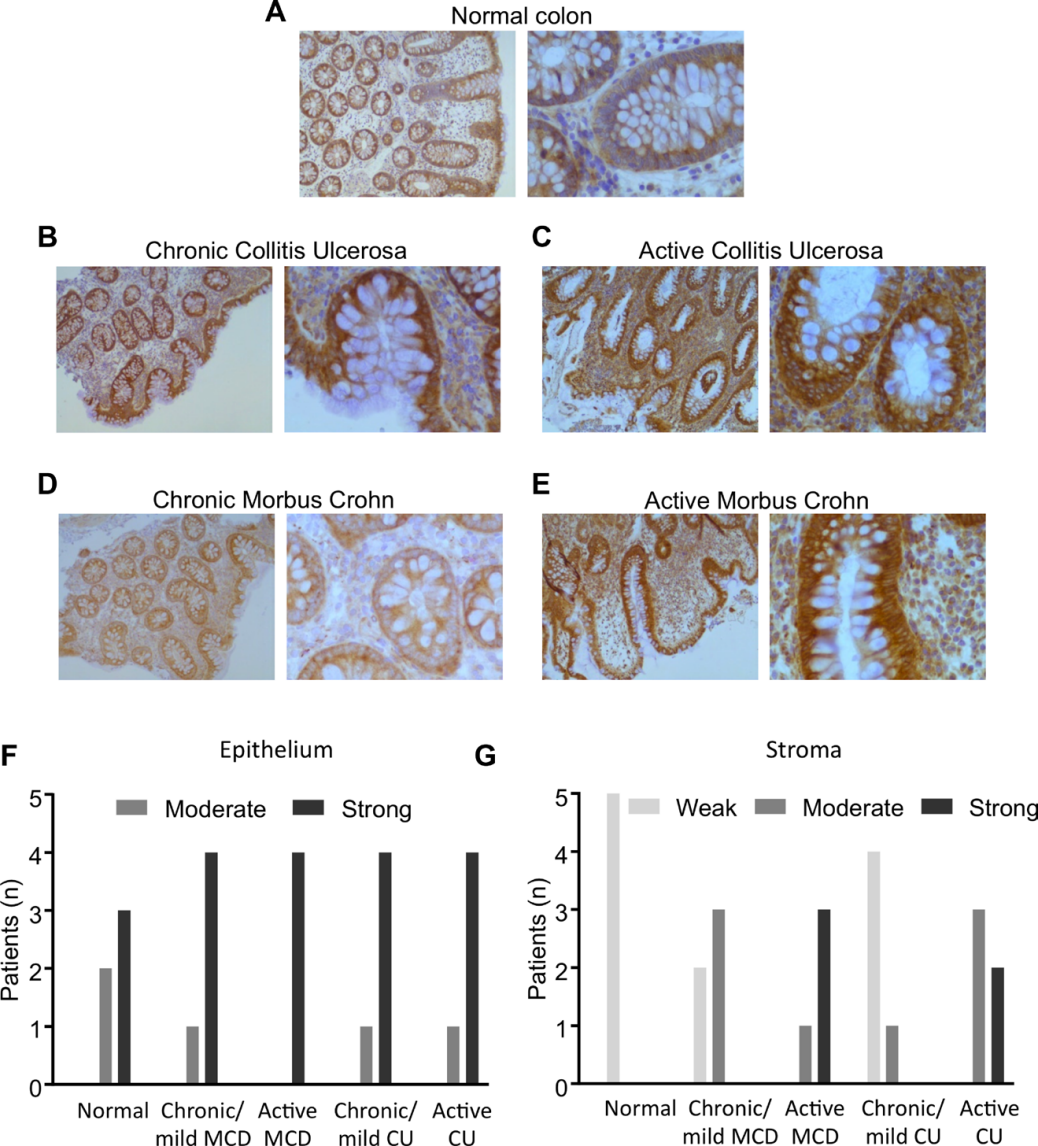
DDX3 expression in colon biopsies Immunohistochemical staining of **(A)** Normal colon (*n* = 5), (**B)** Chronic/mild colitis ulcerosa (*n* = 5), (**C)** Active Colitis ulcerosa (*n* = 5), (**D)** Chronic/mild Morbus Crohn’s disease (*n* = 5), and (**E)** Active Morbus Crohn’s (*n* = 5). (**F)** Epithelial expression of DDX3 in patients with various degrees of IBD. (**G)** Stromal expression of DDX3 in IBD patients.

Using an in-house monoclonal antibody against DDX3, we determined DDX3 expression levels in colonic crypt cells from IBD patient’s colon biopsies. Samples were obtained from normal colon, chronic/mild Colitis Ulcerosa, active Colitis Ulcerosa, chronic/mild Morbus Crohn’s, and active Morbus Crohn’s (Figure 1). Normal colon indicated mild cytoplasmic DDX3 staining in the epithelium with the stroma almost negative. Chronic/mild colitis ulcerosa showed chronically inflamed colonic tissue with crypt fission, distortion and depletion, and with increased inflammatory reaction in the stroma by predominantly plasma cells. Compared to normal colonic tissue, both epithelium and stroma showed increased cytoplasmic staining for DDX3. Active Colitis ulcerosa displayed chronically/actively inflamed colonic tissue with crypt distortion and crypt abcesses, along with increased inflammatory reaction in the stroma by neutrophils and plasma cells. Again, compared to normal tissue, epithelium and stroma showed increased cytoplasmic staining for DDX3. Chronic/mild Morbus Crohn’s disease showed chronically inflamed colonic tissue with mild crypt distortion and increased inflammatory reaction in the stroma by predominantly plasma cells. There was mild cytoplasmic staining for DDX3 in epithelium and stroma. Active Morbus Crohn’s displayed chronically/actively inflamed colonic tissue with crypt fission, distortion and depletion, along with increased inflammatory reaction in the stroma by neutrophils and plasma cells. Compared to normal and mildly inflamed colonic tissue, epithelium and stroma showed increased cytoplasmic staining for DDX3.

Semi-quantitative scoring indicated that, in comparison to normal epithelium, the proportion of the tissues with strong DDX3 expression levels increased in each category of IBD with active CD exhibiting the most prevalent increase (Figure 1F). It was also revealed that, relative to their normal stromal counterparts, the stromal compartments in all IBD cases expressed higher levels of DDX3 and active CD presented with the highest levels of DDX3 (Figure 1G).

### RK-33 can target DDX3 in human colon epithelial cells

**Figure 2:**
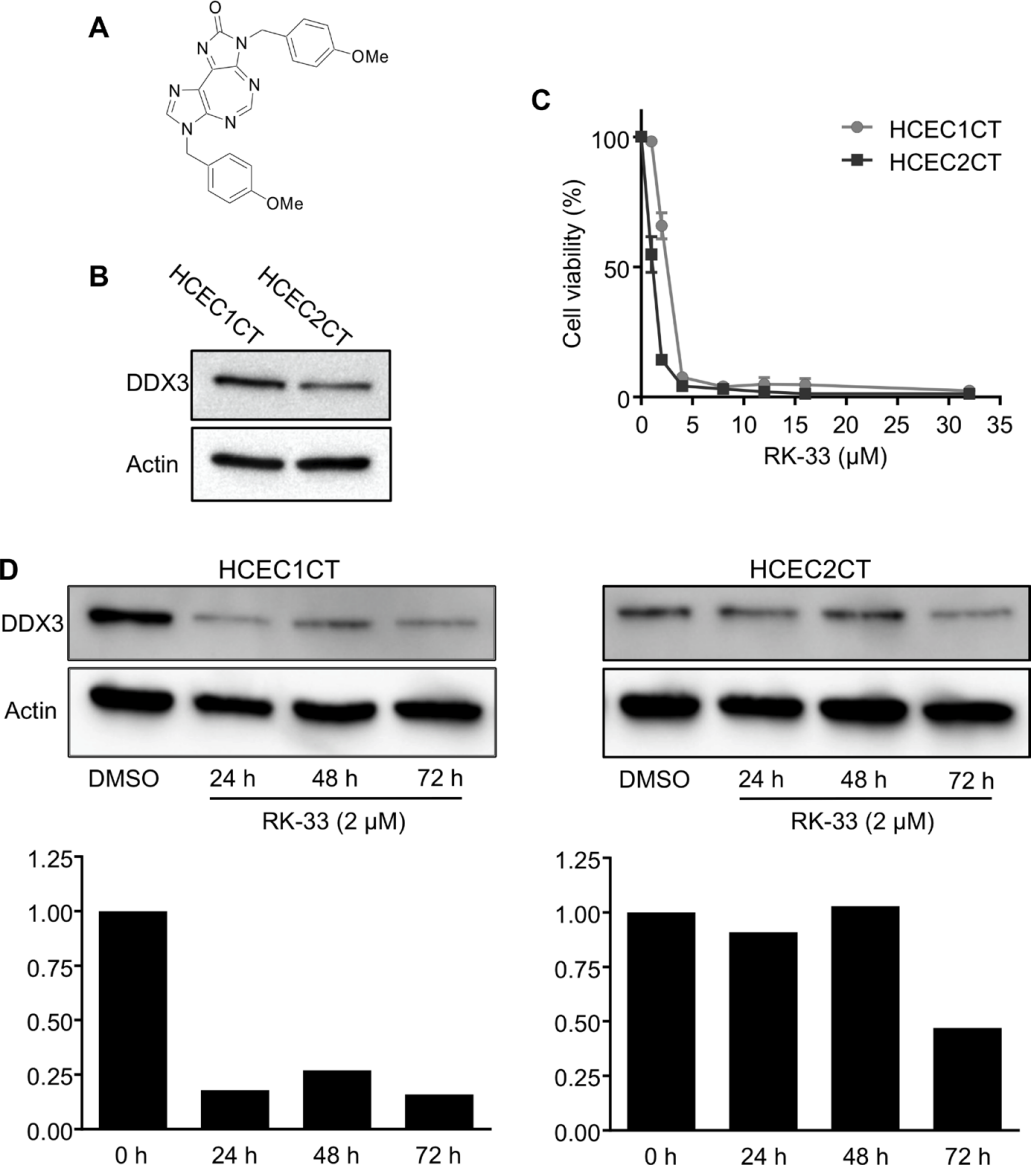
RK-33 sensitivity of human colon epithelial cell lines (**A**) Structure of RK-33. (**B**) Western blot showing DDX3 protein expression levels in HCEC1CT and HCEC2CT cell lines. (**C**) Cytotoxicity assay showing the sensitivity of human colon epithelial cells lines to RK-33. (**D**) Immunoblot showing the expression levels of DDX3 in HCEC1CT and HCEC2CT cell lines treated with RK-33 relative to DMSO as control. Graphs below immunoblots indicate semi-quantitative (relative) expression of DDX3 at various time points.

Our findings that expression levels of DDX3 were directly associated with IBD led us to evaluate DDX3 levels in two human colonic epithelial cells, HCEC1CT and HCEC2CT [25], as a model system to explore the effect of RK-33 treatment on colonic cells. Figure 2A shows the chemical structure of RK-33. As displayed in the western blot of Figure 2B, HCEC1CT cells exhibited higher levels of DDX3 than HCEC2CT cells. Given that DDX3 was present in both cell lines, we tested their sensitivity to inhibition of DDX3 by RK-33. As shown in Figure 2C, HCEC cells were treated with RK-33 at various concentrations ranging from 1 μM to 32 μM or DMSO as a vehicle control. After 72 h of exposure to the drug, cell viability was assessed by MTS assays. HCEC1CT and HCEC2CT cell lines had IC50 values of 2 μM and 1 μM, respectively (Figure 2C). We then determined the DDX3 expression levels in HCEC cells treated with RK-33 or vehicle control after 24 h, 48 h and 72 h exposure. Western blot analysis provided evidence that, relative to the DMSO treated controls, the DDX3 expression levels were reduced in the RK-33 treated HCEC1CT cells at all time points while HCEC2CT cells showed decreased expression after 72 h of RK-33 treatment (Figure 2D).

### RK-33 treatment of HCEC1CT and HCEC2CT cell lines reduces MMP expression

**Figure 3:**
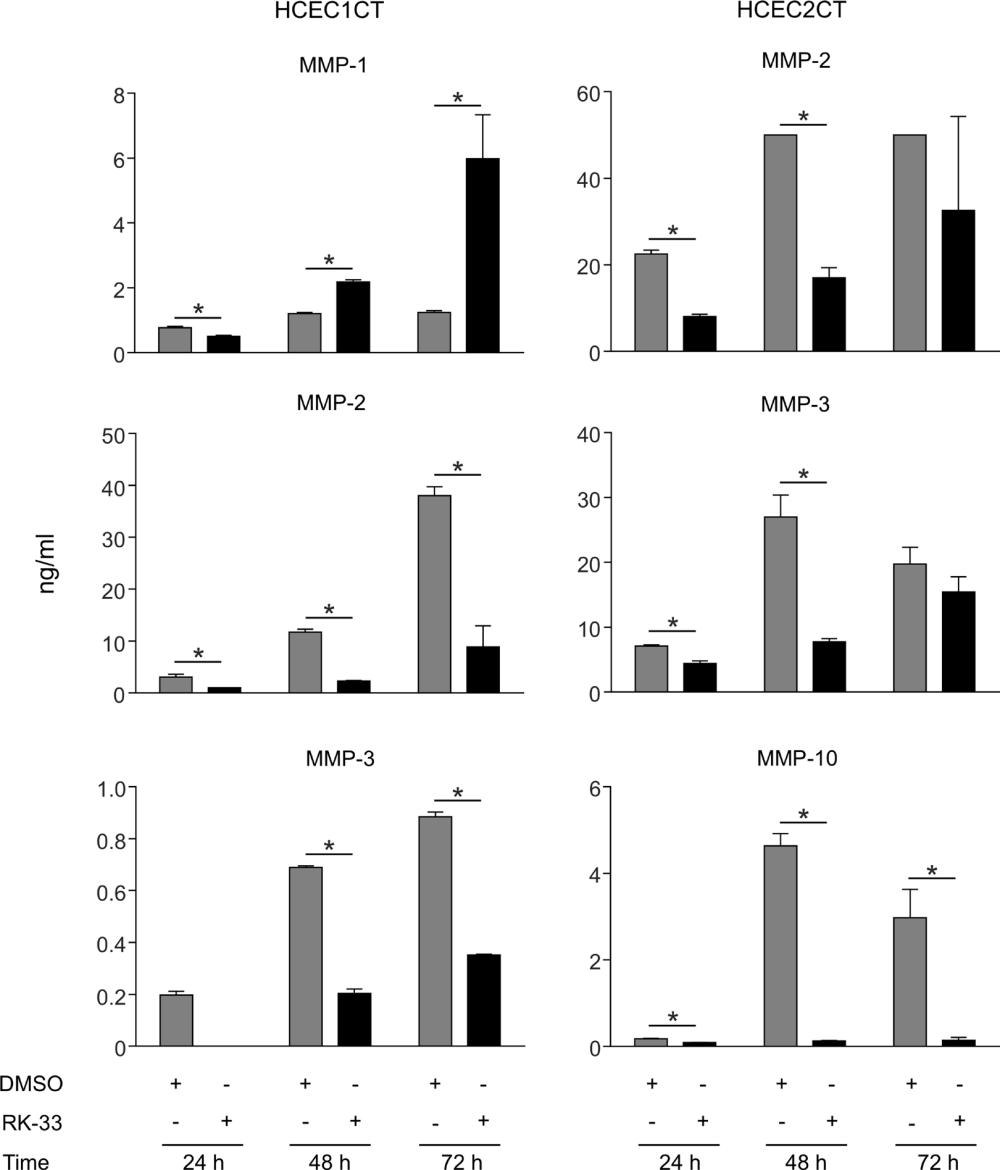
Effect of RK-33 on MMP expression in human colonic epithelial cell lines Supernatants from cell cultures of HCEC1CT and HCEC2CT cell lines treated with vehicle, i.e., DMSO control (light gray bars) or 2 μM RK-33 (dark gray bars) were collected at 24 h, 48 h and 72 h and the indicated MMP concentrations (ng/mL) were determined for both conditions at each point. The experiment was performed twice; error bars represent mean ± SD, ^*^p < 0.05.

Cell culture supernatants were collected after 24, 48, and 72 h of growth from HCEC1CT and HCEC2CT cultures that were treated with RK-33 or DMSO and, MMP-1, MMP-2, MMP-3, MMP-7, MMP-9, MMP-10, MMP-12, and MMP-13 concentrations were estimated. As shown in Figure 3, supernatants from RK-33 treated HCEC1CT and HCEC2CT cell lines exhibited decreases in the expression levels of MMP-2, MMP-3, MMP-10 as compared to the DMSO treated cell lines at all time points, while MMP-1 levels in HCEC1CT supernatants decreased at 24 h and then increased. Given that DDX3 expression is high during active inflammation (Figure 1), these results indicate that targeting DDX3 with RK-33 can reduce the expression levels of several key MMPs involved in inflammation and, as such, provides an indication that targeting DDX3 in IBD patients may be effective at controlling MMP- mediated inflammation.

### Differentiation of normal colon cells leads to decrease in DDX3

**Figure 4:**
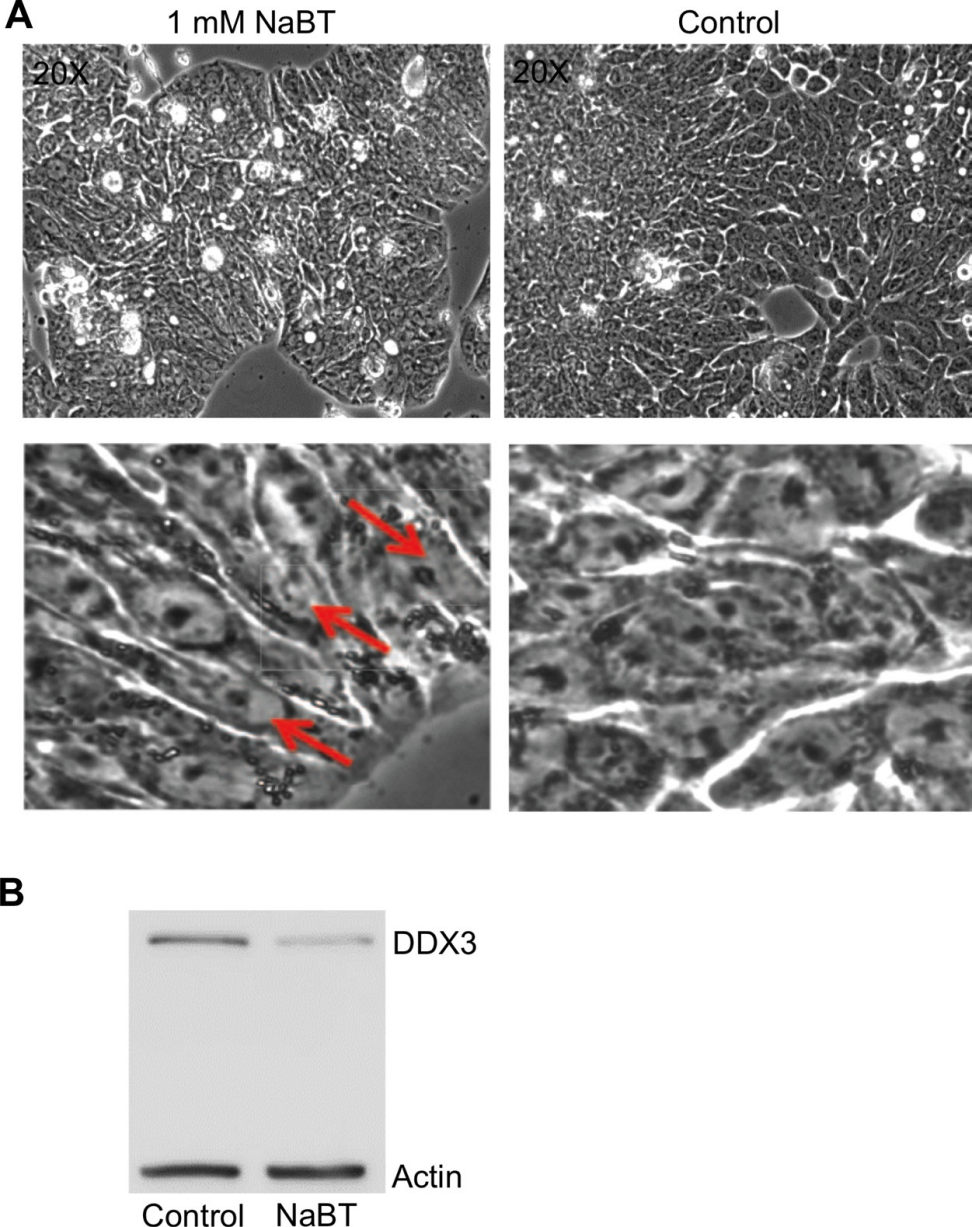
Differentiation of HT29 cells by sodium butyrate (NaBT) results in decreased DDX3 expression (**A**) Photomicrograph images of HT29 cells treated with DMSO (control) or 1 mM NaBT for 96 h. Differentiation to polarized columnar epithelial structures is indicated by red arrows (**B)** Immunoblot showing the levels of DDX3 in the control and NaBT treated cells. Actin was used as a loading control.

To determine if DDX3 protein is an active component of promoting/sustaining an undifferentiated state, i.e, precancerous or cancerous state, HT29, a human colonic cancer cell line that is undifferentiated in standard culture conditions [26, 27], was treated with sodium butyrate to facilitate differentiation. As can be seen in Figure 4A, HT29 cells undergo differentiation following sodium butyrate (NaBT) treatment (1 mM for 96 h). At higher magnification of the upper images of Figure 4A, one can readily observe the appearance of polar columnar aligned cells as an indication that differentiation occurred in the NaBT treated cells (Figure 4A: lower left image), as opposed to the disorganized undifferentiated state of the untreated cells (Figure 4A). Figure 4B indicates that DDX3 levels were significantly lower in the differentiated cells as compared to the undifferentiated cells. This result indicates that DDX3 may be a contributing factor in sustaining an undifferentiated state and as such, it’s potential utility as a biomarker for the progression of IBD towards an undifferentiated cancerous state.

## DISCUSSION

IBDs, which include UC and CD, are chronic inflammatory diseases of the gastrointestinal tract. Different mediators are responsible for the development of this inflammation. Among such mediators, MMPs are important components in the development of chronic inflammation in IBD. MMPs are endopeptidases that mediate degradation of all components of extra-cellular matrix [28] and previous reports have shown differential gene expression of MMPs in the inflamed mucosa of patients with IBD in comparison to uninvolved mucosa [29]. As such, an imbalance between MMPs and their natural tissue inhibitors, in a manner that favors increased MMP activity, contributes to pathogenesis of IBD [29–31]. T-cells and TNF-α [31–34] induce MMP-3 and MMP-10 activity causing tissue degradation in the inflamed intestine and increased levels of these enzymes are found in mucosal samples from patients with IBD [29, 30, 35, 36]. Overall, the severity of inflammation directly correlates with the over expression of MMP-1 and MMP-3 and has been reported in ulcerated and inflamed areas of colon mucosa of UC patients [36]. Consequently, infliximab, a chimeric monoclonal antibody against TNF-α, has become a mainstay of therapy in IBD [37]. Upregulation of MMP-2 in pediatric CD has also been reported [38]. Therefore, given that MMP-1, MMP-2, MMP-3, MMP-7, and MMP-10 have definitive contributions to the mucosal damage in IBD patients, a consideration of targeted MMPs treatment strategies is warranted in IBD [39].

It’s important to take a note that patients with severe and prolonged IBD have a high risk of developing colorectal cancer (CRC) and chronic inflammation is a major contributing factor that predisposes these patients to the development of this cancer. CRC is the third most prevalent cancer in men and women and is also the third leading cause of cancer-related deaths [40]. Upregulation of MMPs in CRC tissue samples has been reported often [41, 42], and the over expression of MMP-1, MMP-2, MMP-3, MMP-7, MMP-9, and MMP-13 has been associated with worse outcome and poor overall survival, which is an indication that MMPs could be potential targets for the treatment of CRC [22, 23, 43]. As presented in this study, treating human colonic epithelial cells with RK-33 results in the downregulation of MMP-1, MMP-2, MMP-3, and MMP-10 expression. This indicates yet another regulatory function of DDX3 to add to the several others that have been revealed in our studies of this RNA helicase [44].

We have previously reported the increased expression of DDX3 in 303 colorectal cancers by immunohistochemistry and its functional involvement in CRC [45]. During these studies, we reported that–RK-33, specifically targets DDX3 and abrogates its function in cells with elevated DDX3 expression levels and, as such, has a potential to be a drug that can target a subset of CRC patients [45]. To determine if DDX3 could be a contributing factor in IBD, we conducted immunohistochemistry on colon biopsies. The results indicated that expression levels of DDX3 was increased relative to normal tissue in both the epithelium and stromal compartments in IBD cases with the greatest increases occurring in those cases with active inflammation. This is a novel finding in our continuing identification of the functions of DDX3 in diseased tissues [44–46] and we thus carried out the present studies to begin to explore in what ways an induction of DDX3 expression within inflamed CD and UC tissues could be contributing to the inflammatory processes of these disease.

In conclusion, similar to infliximab treatment in CD patients, which reduces the serum expression levels of MMP-1, MMP-2, and MMP-3 [47, 48], RK-33 treatment has the potential of generating a comparable result by targeting DDX3 and lowering the expression levels in and their inflammatory contributions to IBD pathogenesis. In addition, from the differentiation assay using sodium butyrate treatment, we demonstrated that DDX3 could have a function in sustaining an undifferentiated state, which is characteristic of transformed colonic tissue. Consequently, targeting DDX3 in IBD with RK-33 also affords the possibility of preventing colorectal cancer, especially in high-risk patients with longstanding and extensive involvement of IBD.

## MATERIALS AND METHODS

### Tissue culture

Human colon epithelial cells, HCEC1CT and HCEC2CT cells were grown in DMEM media supplemented with Hyclone 199 media with EBSS (Thermo Fischer Scientific, MA), 2% Hyclone cosmic calf serum (Thermo Scientific, MA), EGF (20 ng/mL, Sigma-Aldrich, Milwaukee), hydrocortisone (1 μg/mL, Sigma-Aldrich, Milwaukee), insulin (10 μg/mL, Thermo Fischer Scientific, MA), apotransferin (2 μg/mL, Sigma-Aldrich, Milwaukee), sodium selenite (5 nM). HT29 cells were grown in McCoy’s 5A medium supplemented with 10% fetal bovine serum.

### Patient samples

Colon biopsy samples were selected from formalin fixed paraffin embedded tissue retrieved from the archives of the UMC Utrecht, The Netherlands. Colon tissue with no abnormalities was compared to colon tissue with signs of chronic or active Morbus Crohn and chronic or active ulcerative colitis. Each group contained five patients. For this study, only anonymous archival pathology material was used. Therefore, no informed consent is required according to Dutch legislation, as this use of redundant tissue for research purposes is part of the standard treatment agreement with patients in the UMC Utrecht.

### Immunohistochemistry

Four micron thick sections were cut, mounted on Surgipathe X-tra adhesive slides (Leica Biosystems, Milton Keynes, UK), deparaffinized in xylene and rehydrated in decreasing ethanol dilutions. Endogenous peroxidase activity was blocked with 1.5% hydrogen peroxide buffer for 15 minutes and was followed by antigen retrieval by boiling for 20 minutes in EDTA buffer (pH 9.0). Slides were blocked with protein block from Novolink Polymer Detection System (Leica Microsystems, Eindhoven, The Netherlands) and subsequently incubated in a humidified chamber for 1 hour with monoclonal anti-DDX3 antibody at 1:50 dilution [49]. Post primary block, secondary antibodies and diaminobenzidine treatment were performed with the same Novolink Polymer Detection System according to the manufacturer’s instructions. The slides were counterstained with hematoxylin and mounted. Appropriate positive and negative controls were used throughout the experiment.

Scoring was performed by consensus of two observers (Paul van Diest. and Marise Heerma van Vos). DDX3 expression was observed in both the epithelial and stromal compartment. The intensity of cytoplasmic DDX3 expression in both compartments varied and were therefore scored semi-quantitatively as absent (0), weak (1), moderate (2), or strong (3). Cases with score 0 to 2 were classified as having low DDX3 expression while cases with score 3 were classified as having high DDX3 expression, as reported previously [45].

### Differentiation assay using sodium butyrate treatment

HT29 cells were plated at 1 × 10^5^ cells in a 6-well plate. After over-night incubation, cells were treated with 1 mM and 2.5 mM sodium butyrate (NaBT) for 48 h, 72 h, and 96 h.

### Immunoblotting

HCEC1CT and HCEC2CT cells were plated at 1.5 × 10^5^ cells per well in a 6-well plate and incubated overnight for attachment. Cells were treated with DMSO or 2 μM RK-33 for 24, 48 and 72 h and lysed in SDS-extraction buffer (100 nM Tris-HCl, 2% SDS, 12% glycerol, 10 mM EDTA, pH 6.7) with added protease inhibitor. Protein concentration was determined and 30 μg of each sample was loaded on-to 10% SDS-PAGE gels. Proteins were transferred to PVDF membranes, the membranes were blocked in 5% skim milk for 1 h and then primary antibodies: anti-DDX3 (1:500 in 5% BSA) and anti-actin (1:10,000 in 5% skim milk) were added followed by over-night incubation at 4°C. After three washes with TBST, horse radish peroxidase conjugated-mouse secondary antibodies were added and incubated at room temperature for 1 h. The blots were developed with Clarity Western ECL (BioRad, Hercules, CA, USA) and imaged with G:Box Chemi XR5 (Syngene, Frederick, MD, USA).

### Cytotoxicity assay

Cytotoxicity was determined using MTS assays. HCEC1CT and HCEC2CT cells were plated in triplicates at 1 × 10^3^ cells per well in a 96-well plate. After over-night incubation, cells were treated with various concentrations of RK-33 (1–32 μM) and DMSO as a control. After 72 h of incubation, 10% MTS reagent was added, plates were returned to the cell culture incubator for an additional 2 h, and absorbance was determined at 490 nm.

### Detection of MMPs from RK-33 treated HCEC1CT and HCEC2CT cell lines

HCEC1CT and HCEC2CT cell lines were plated at 5 × 10^5^ cells in 100mm dish. After overnight incubation, cells were treated with RK-33 (2 μM) and DMSO as a control. Supernatants were collected after at 24, 48 and 72 h. MMP levels in tissue culture supernatants were measured on the Bio-Plex 200 suspension array system (Biorad, Hercules, CA) using the Millipore panels HMMP1MAG-55K (MMP-3, MMP-12, MMP-13), HMMP2MAG-55K (MMP-1, MMP-2, MMP-7, MMP-9, MMP-10) following the vendor protocols. The concentrations (ng/ml) of target molecules were determined using the Bio-Plex manager software.
